# A retrospective study on application of fibula/iliac flap surgical techniques to mandibular defects

**DOI:** 10.1038/s41598-023-43643-4

**Published:** 2023-10-02

**Authors:** Ning Gao, Kun Fu, Jinghua Cai, Wei He

**Affiliations:** Department of Oral and Maxillofacial Surgery, First Affiliated Hospital of Zhengzhou University, Zhengzhou, 450052 China

**Keywords:** Medical research, Oral diseases

## Abstract

This study group consists of a total of 61 patients who underwent fibula flap and iliac flap surgeries to repair mandibular defects. Patients’ Quality Of life (QOL) at 6 and 24 months after surgery is investigated and compared by the EORTC-QLQ-H&N and OHIP-14. The base data of the two groups of patients are collected and analysed by the SPSS 20.0 statistical software. Independent sample t test was conducted for EORTC-QLQ-H&N and OHIP-14 scores at two time points in each group. The 61 cases of free flap all survived and the difference in the location of the primary tumor between the two groups is statistically significant. The EORTC-QLQ-H&N showed that the score of speech, diet, social contact, and teeth all went up at 6 months after surgery, but went down dramatically at 24 months after surgery. The OHIP-14 showed that there was significant reduction in functional limitation at 24 months after surgery, with statistical significance (p < 0.05) between the groups of iliac flap (19.16 ± 5.33) and fibula flap (33.77 ± 7.71). Therefore, it is suggested that patients suffering from mandibular defects receive surgery utilizing the iliac flap, while those with a larger range of defects or lesions involving the condyle and chin should receive corrective surgery utilizing the fibular flap.

## Introduction

Ameloblastoma is a common tumor of the mandible, and extended mandibular resection can effectively prevent tumor recurrence. However, the jaw defect caused by tumor resection has a serious impact on the patient’s facial shape, bite and chewing functions, which not only interferes with the patient’s social activities, but also increases their psychological burden^1^. There are three major challenges faced by mandibular defects caused by ameloblastoma. The first challenge is how to restore the continuity of the mandible and maintain the patient’s maxillofacial shape. The second challenge is how to restore the integrity of the dentition and restore the patient’s chewing and language functions. The third challenge is how to reduce the emotional damage of the disease to the patient, so that they can obtain a better quality of life. Since Hidalgo reported the successful repair of mandibular defects with fibular free flaps in 1989, the clinical application of free flaps has become more and more mature^2^. With the gradual maturity of digital and microsurgical techniques^3^, the jaw is repaired by iliac flaps^4^ and fibular flaps^5^ in more and more cases. With the gradual improvement of implant materials and repair technology^6^, implant repair on grafted bone can better restore the patient’s occlusal and chewing functions^7^, and further improve the patient’s QOL^8^. The study has adopted the 14-item Oral Health Impact Profile questionnaire (OHIP-14)^9^ and the European Organization for Research and Treatment of Cancer Quality of Life Questionnaire Head and Neck (EORTC-QLQ-H&N)^10^ to evaluate and analyze the QOL of patients with mandibular ameloblastoma repaired by iliac flap and fibular flap. It provides the basis to develop comprehensive treatment plan, select surgical methods and repair methods, as well as choose postoperative functional rehabilitation and possible psychological intervention for patients with mandibular ameloblastoma.

## Results

All 61 free fibular flaps survived, and 3 patients with fibula repaired mandibles had discomfort in the temporomandibular joint area, but no obvious tenderness during postoperative re-examination. The degree of opening of all patients ranged from 3.1 to 3.9 cm. The osseointegration of the implants was good, and the occlusal relationship was restored. The natural curvature, height and occlusal relationship of the mandibular defect area of all patients were recovered, without obvious abnormality in the operation area. There were no obvious complications in the donor site. The lower extremities can bear weight, and walking is not affected, without obvious dysfunction of the donor site. All 61 patients completed the survey, and the completion rate was 100%. The general situation is shown in Table 1.

**Table 1:** Details of patients.

Variables	Total patients (%)	Iliac (n = 23)	Fibula (n = 38)	*p* value
Age (years)				0.304
< 50	54	21	33	
50 and over	7	2	5	
Sex				0.115
Male	29	11	18	
Female	32	12	20	
Primary sites of tumor				0.000
Body	26	20	6	
Body, angle	21	3	18	
Body, angle, ramus	14	0	14	
Clinical type				0.586
Solid	57	21	36	
Unicyclic	4	2	2	
Radiographic appearance				0.252
Unilocular	8	4	4	
Multilocular	53	19	34	

### EORTC-QLQ-H&N survey results

The specific scores of EORTC-QLQ-H&N are shown in Table 2. The scores of speeches, diet, social contact and teeth were higher at 6 months after surgery, and decreased significantly at 24 months after surgery, with statistically significant difference (p < 0.05). There was no difference between the two groups. The scores of mouth opening and swallowing were both lower at 6 months and 24 months after surgery, without statistically significant difference (p > 0.05). There was no difference between the two groups.

**Table 2:** Means of scores of items and scales of EORTC-QLQ-H&N Questionnaire.

Domains	Vascularized iliac flap			Vascularized fibular flap			*t *(*p*) value (24 months)
	6 months	24 months	*t *(*p*) value	6 months	24 months	*t *(*p*) value	
Swallowing	24.65 ± 10.77	23.17 ± 5.91	0.577 (0.283)	28.71 ± 10.23	28.53 ± 9.25	0.082 (0.688)	2.481 (0.069)
Speech	45.26 ± 14.24	27.13 ± 3.71	5.909 (0.000)	38.79 ± 13.97	30.24 ± 9.30	3.142 (0.000)	1.526 (0.033)
Open mouth	23.17 ± 7.23	22.96 ± 6.54	0.107 (0.440)	30.42 ± 11.61	32.47 ± 11.49	0.775 (0.894)	3.626 (0.124)
Diet	43.52 ± 13.44	26.26 ± 3.51	5.961 (0.000)	40.37 ± 13.72	29.45 ± 9.55	4.027 (0.000)	1.535 (0.013)
Social contact	53.26 ± 11.61	25.83 ± 5.10	10.379 (0.003)	52.79 ± 14.04	29.45 ± 9.88	8.380 (0.009)	1.628 (0.053)
Teeth	58.48 ± 12.93	25.39 ± 5.32	11.351 (0.008)	60.68 ± 17.28	30.95 ± 9.99	9.182 (0.007)	2.459 (0.078)

### OHIP-14 survey results

The specific scores of OHIP-14 are shown in Table 3. There was no difference in physical pain before and after surgery (p > 0.05) between the two groups. The scores of psychological discomfort, psychological disability and social disability were the highest at 6 months after surgery. The scores of psychological discomfort and psychological disability both decreased at 24 months after surgery, without statistically significant difference compared with that at 6 months after surgery (p > 0.05). The scores of physical disability, handicap and social disability significantly reduced at 24 months after surgery, and the difference was statistically significant compared with that at 6 months after surgery (p < 0.05). The scores of functional limitations significantly reduced in both groups at 24 months after surgery. However, the iliac bone group (19.16 ± 5.33) was still statistically significant compared with the fibula group (33.77 ± 7.71) (p < 0.05).

**Table 3:** Means of scores of items and scales of OHIP-14 Questionnaire.

Vascularized iliac flap				Vascularized fibular flap			*t *(*p*) value (24 months)
Domains	6 months	24 months	*t *(*p*) value	6 months	24 months	*t *(*p*) value	
Functional limitation	68.25 ± 14.63	18.04 ± 5.30	15.508 (0.000)	64.55 ± 15.35	30.59 ± 9.46	11.497 (0.030)	5.806 (0.008)
Physical pain	29.21 ± 6.39	22.61 ± 9.48	2.811 (0.091)	29.03 ± 9.17	24.24 ± 7.40	2.482 (0.156)	0.746 (0.124)
Psychological discomfort	73.83 ± 12.22	49.87 ± 13.18	6.469 (0.666)	66.82 ± 16.37	45.68 ± 14.31	5.948 (0.328)	1.137 (0.735)
Physical disability	57.54 ± 24.07	24.13 ± 8.51	6.288 (0.000)	60.76 ± 21.20	24.70 ± 7.08	9.826 (0.000)	0.281 (0.056)
Psychological disability	70.17 ± 13.28	49.57 ± 12.93	5.385 (0.691)	67.63 ± 14.89	46.51 ± 14.55	6.211 (0.811)	0.823 (0.567)
Social disability	74.79 ± 12.48	31.96 ± 6.90	14.471 (0.010)	70.79 ± 12.56	22.95 ± 6.45	20.672 (0.000)	5.125 (0.893)
Handicap	54.21 ± 23.00	27.61 ± 9.13	5.168 (0.000)	57.61 ± 20.59	26.59 ± 7.56	8.612 (0.000)	0.466 (0.069)

## Discussion

Ameloblastoma is one of the most common tumors of the mandible. Extended mandibular resection can effectively prevent recurrence, but the bone defect after resection directly affects the patients’ facial, chewing and pronunciation functions^11^. At the same time, it also seriously affects the patients’ daily life and social activities, causing damage to the patients’ psychology. With the maturity of microsurgery technology and the rapid development of digitization^12^ and 3D printing technology^13^, iliac bone flap and fibula flap have been widely used in clinical practice as precision medical technology. With the improvement of implant materials and repair technology^14^, implant repair after bone transplantation has been promoted^15^. With the transformation of the biomedical model, in addition to the biological indicators such as survival rate and organ function reconstruction, the standard for evaluating the effect of tumor treatment also aims to make patients get better QOL^16,17^.

### Popularization of vascularized iliac bone flap and vascularized fibular flap in clinical practice as precision medical technology

With the development of digital technology and microsurgery technology, fibular flap^18^ and iliac flap^19^ are widely used in mandibular defect repair and reconstruction. The study has adopted preoperative digital technology to simulate diseased mandibular resection of all patients. It then has employed mirror technology to restore the shape of the diseased mandible, and undertaken fibula or ilium osteotomy. Afterwards, it has pre-formed the titanium plate on the head mold which was printed by 3D printing technology. During the surgery, the transplanted bone is shaped according to the titanium plate, which greatly shortens the time and improves the accuracy. It also perfectly restores the shape of the mandible and avoids facial collapse and occlusal disorder caused by jaw defect.

**Figure 1:**
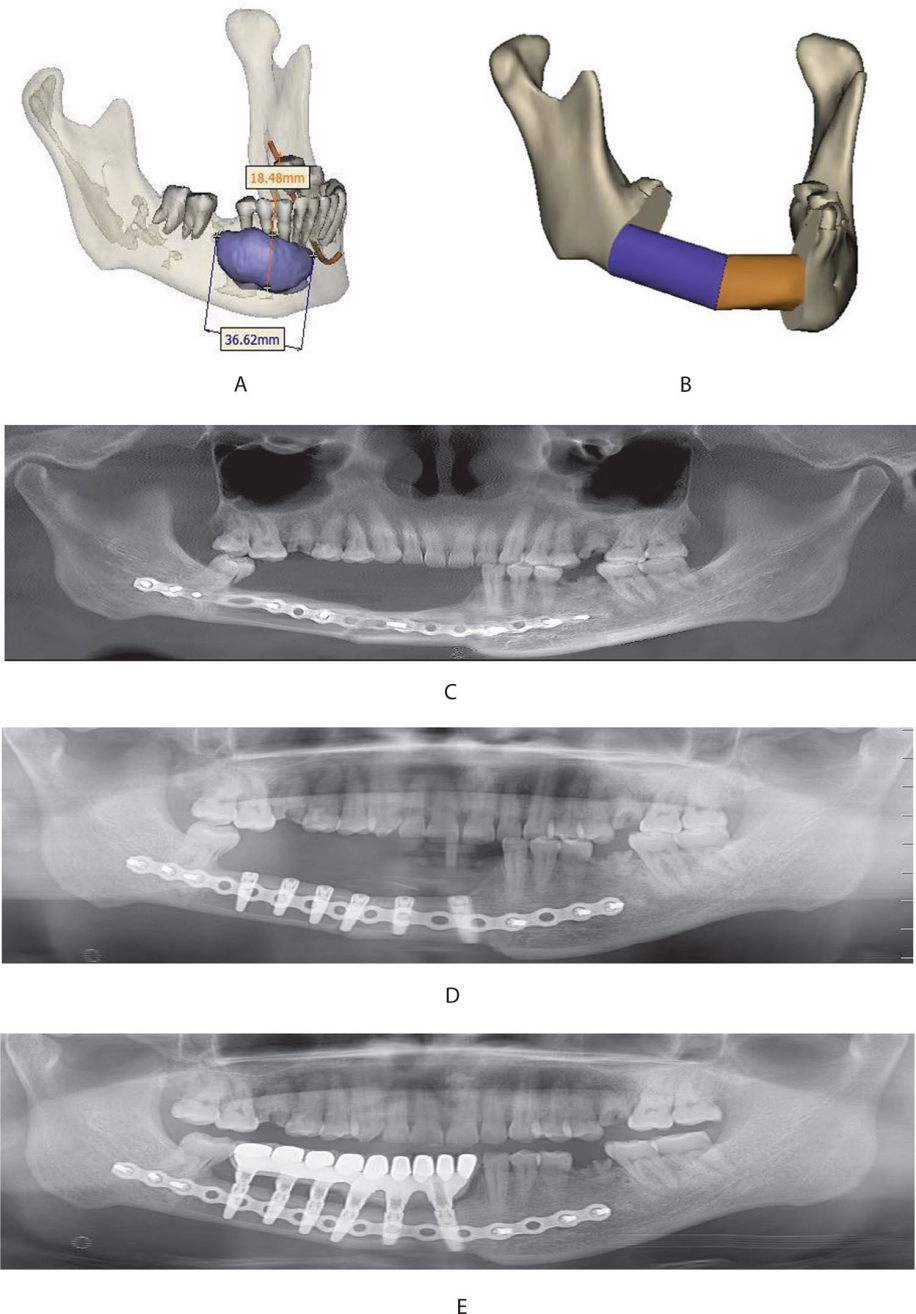
(**A**) Patients: male, 48 years old, with an ameloblastoma of the right mandible and the lesion being located in the body of the right mandible; (**B**) the mandibular resection area was simulated and fibula was used for reconstruction; (**C**) the mandibular defect was repaired by resecting the diseased mandible and folding fibula; (**D**) implant placement; (**E**) the patient's chewing function was restored by placing the implants.

**Figure 2:**
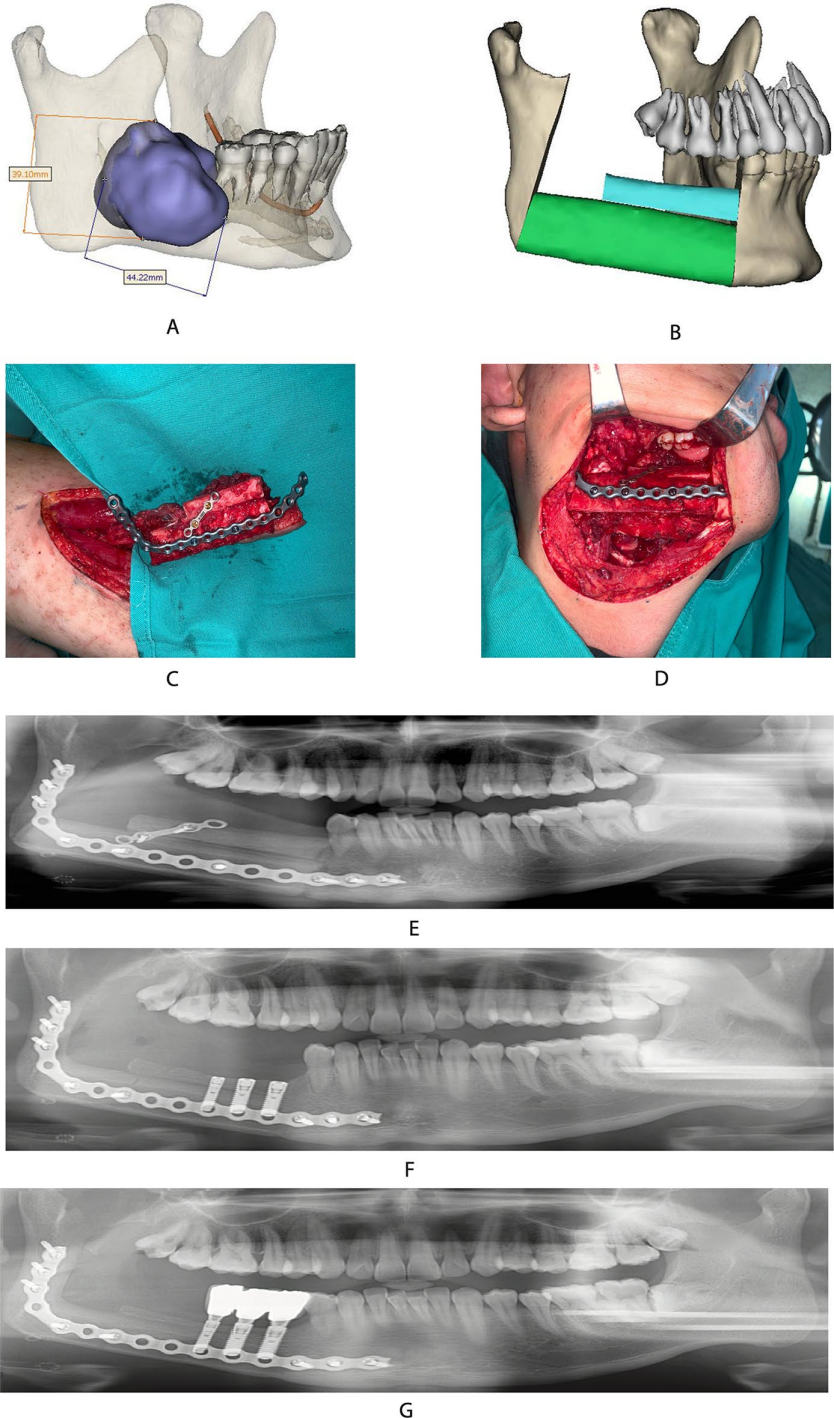
(**A**) Patients: female, 18 years old, with ameloblastoma of the right mandible and the lesion being located in the body of the right mandible; (**B**) digital setting of fibula restoration range; (**C**) shaping the fibula based on the pre-formed titanium plate; (**D**) double-layer fibula for mandibular repair; (**E**) the patient's occlusal relationship was better maintained after the fibula was restored; (**F**) implant placement at 6 months after fibula placement; (**G**) the patient's chewing function was restored by placing the implants. There was no obvious peri-implant inflammation at 60 months follow-up visit after implant placement.

The fibular flap is a long tubular bone dominated by cortical bone with sufficient bone mass. The resectable fibula length is 20–26 cm. In this group of cases, the length of cut is 16–24 cm, which can meet a wide range of bone defects. Moreover, the fibula has a segmental blood supply from the periosteum and an intraosseous blood supply from the nourishing artery, making it plastic and more conforming to the shape of the mandible^20,21^. However, the height of the fibula is 1.3–1.5 cm. Hence the repair of mandibular defects with single-layer fibula faces the problem of crown-to-root ratio imbalance and peri-implantitis^22^ (Fig. 1), which can only be solved by folded fibula (Fig. 2).The fibula is a type I bone, with almost no cancellous bone except for the cortex and medullary cavity. The whole process of tapping is required to place the implant during the operation.

**Figure 3:**
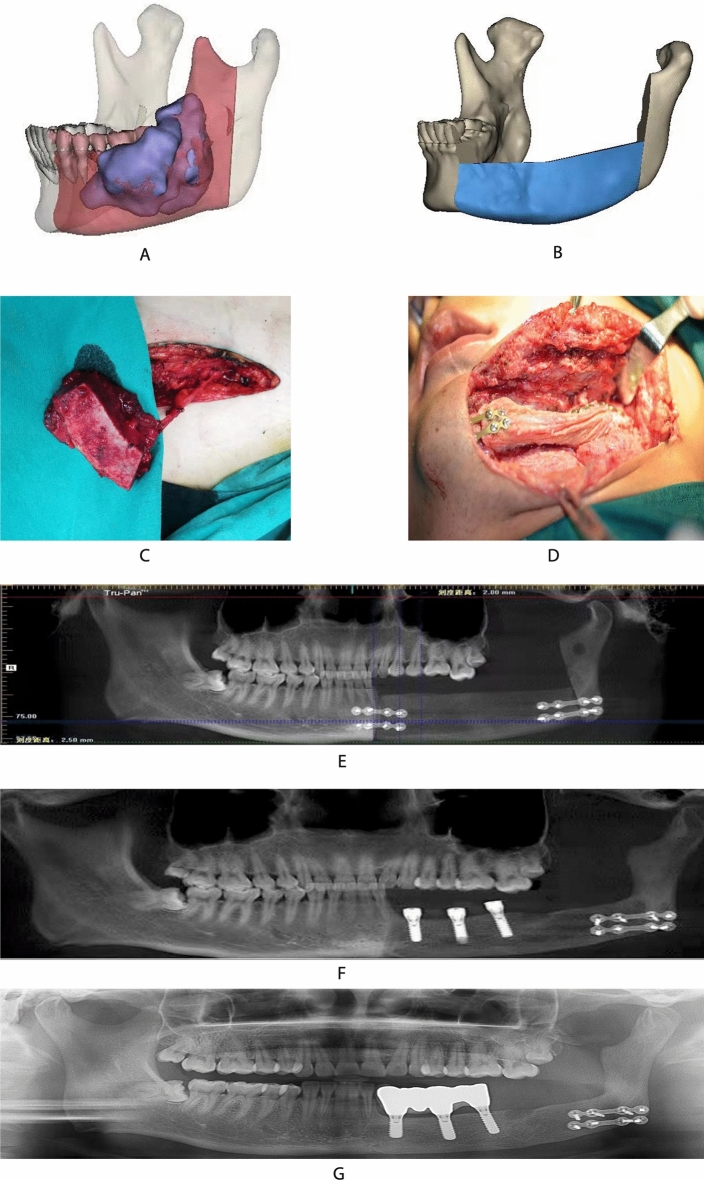
(**A**) Patient: male, 38 years, old with ameloblastoma of the left mandible. It shows the location of the lesion and the scope of resection; (**B**) the ilium is simulated to restore the mandible; (**C**) preparation of vascularized ilium; (**D**) the diseased mandible is removed, and the ilium is used to repair and reconstruct; (**E**) the curved layer shows the mandible range repaired by the ilium; (**F**) implant placement; (**G**) the repair was completed. There was no obvious peri-implant inflammation at 36 months follow-up visit after implant placement.

The iliac bone flap is cancellous bone^23^. Hence, the ilium cannot be shaped arbitrarily even though the longest donor bone can be up to 14 cm in length. It is not suitable to reconstruct the bone defect with a long span^24^. Likewise, for lesions involving the condyle and chin, the fibula is recommended for repair. The ilium is a type II–III bone, with large bone fragments. The reconstructed mandible can have a certain height and width, which is more suitable for implant placement (Fig. 3). However, to Compared with the fibula, the ilium’s anti-infection ability in the later stage is not as good as that of the fibula. However, it is relatively concealed and has fewer side effects on the lower extremities^25^.

Here are precautions in mandibular defect reconstruction: (1) In mandibular resection, it is often necessary to strip or even remove the medial pterygoid and masseter muscles. The reattachment of the masticatory muscle cannot completely restore its biomechanical properties to normal, but will have a certain impact on the mandibular opening and closing movement and chewing efficiency. Therefore, excessive stripping of the masticatory muscles should be avoided. (2) The function of cheek and tongue can improve chewing efficiency by sensing and transporting food. Therefore, under the premise of not violating the principle of no tumor, the buccal and tongue tissues and their sensorimotor nerves should be protected as much as possible. (3) In order to avoid the rotational torsion of the ilium during chewing, it is recommended to choose a four-hole titanium plate rather than the reconstruction plate alone for fixation of the vascularized iliac bone. The fibular flap is recommended to be fixed with a reconstruction plate, while the folded part is recommended to be fixed with a four-hole titanium plate or a lag screw.

### Necessity to implant restoration

The purpose of repairing and reconstructing mandibular defect is to restore its function and shape, so implant denture repair is an important part of the reconstruction process. Implant restoration can better recover the integrity of the dentition^26^ as well as patients’ occlusal and masticatory functions, hence improve their QOL^15^. Here are precautions for Implant Restoration: (1) Partial implants are mostly chosen to support the restoration of fixed dentures. However, when the defect range is large and the number of implants is not enough to support the restoration of fixed dentures, or when the vertical distance is large and the overbite coverage relationship is special, overdentures can be selected for restoration. (2) Screw retention is recommended for the upper porcelain crown. Once peri-implantitis occurs, it is convenient to remove it in time. All patients in this group were retained with screws. (3) Patients must be able to use dental irrigators and interdental brushes for periodontal maintenance. They must return for follow-up visits on time, hence get any possible problems such as peri-implantitis and occlusal interference dealt with in a timely manner. (4) The CBCT should be regularly took to understand the bone resorption around the implant. In addition, peri-implantitis remains the leading cause of implant loss in bone grafts. More research is required to reduce the occurrence of peri-implantitis.

### Influence of various indicators of QOL on patients

With the development of medical technology, repair and reconstruction after ameloblastoma excision can well restore the patient’s facial shape and chewing function, hence minimize the damage caused by the disease to the patient. However, the psychological impact of disease and surgery on patients lasts for a long time. How to further improve the QOL of patients has become the common goal of doctors and patients^27^.

EORTC-QLQ-H&N35 mainly investigates various functions of the oral cavity. The patient’s swallowing function was not affected because the lingual and hypoglossal nerves were not damaged during the surgery. Some patients had slurred speech due to tooth loss. however, it gradually returned to normal at 24 months after surgery. The scores diet, social contact, and teeth were highest at 6 months after surgery. The scores gradually returned to preoperative levels as implants were placed and porcelain crowns were restored. The score of the fibula group was slightly lower than that of the ilium group at 24 months after surgery. Patients with single-layer fibula repairing mandibular defects are still afraid to chew on the affected side. Overall, patients were very satisfied with their facial shape, occlusal relationship and chewing function.

OHIP-14 conducted a comprehensive investigation of the patients from the physiological and psychological aspects. After fibula transplantation, although the integrity of the mandible was restored, the loss of teeth and the decline of masticatory function still had a great impact on the patient’s psychology.

All patients had completed dental implant restoration at 24 months after surgery, and their occlusal relationship and masticatory function were gradually restored. The scores of physical impairment, disability and social impairment were significantly lower at 24 months after surgery, with statistically significant difference compared with that at 6 months after surgery. There was no difference between the two groups. It shows that with the implant placement, patients were very satisfied with their facial shape, occlusal relationship and chewing function. Functional limitation was significantly lower in both groups at 24 months after surgery, but remained statistically significant in the ilium group compared with the fibula group. Here are three main reasons for consideration: (1) Patients in the fibula group often involved the ascending ramus of the mandible, and most of the condylar needed to be removed during the surgery. Patients feel discomfort in the joint area after surgery. (2) In the fibula group, a single layer of fibula should be selected to repair the mandibular defect if the defect range is large. It will lead to crown-to-root ratio imbalance and the occurrence of peri-implantitis. (3) The lack of fibula leads to the inability of long-term weight bearing of the lower limb on the affected side, patients will feel tired and weak after the activity. The scores of psychological discomfort and psychological disorder both decreased at 24 months after surgery, without statistically significant difference compared with that at 6 months after surgery. There was no difference between the two groups. Patients remain concerned about tumor recurrence, suggesting that recovery from psychological trauma is much slower than recovery from physical lesion. Foreign scholars have provided psychological counseling to patients with maxillofacial tumors, and achieved good result. Psychological problems in cancer patients have also attracted the attention of clinicians in China. While treating the disease, the fear and anxiety of patients is designed to rescued by well informed of the disease knowledge before surgery. Postoperative discomfort in the oral, maxillofacial and lower limbs needed to be diagnosed and treated in a timely manner, and patients were encouraged to actively participate in social activities. The survey has also showed that the encouragement and support from family members has significantly beneficial to help patients recover from the trauma of the disease, which is important for their physical and mental health of patients. Therefore, joint efforts by medical staff, patients and their family members are all required towards alleviating the psychological discomfort and social barriers of patients. It is necessary to establish contact with professional psychologists and use more professional means to conduct objective tests, in order to more fully address the psychological impact of the disease on patients during subsequent treatment. Considering the prominent advantages the fibula graft brings to the reconstructive facial surgery, it is reasonable to have complications, namely the comparatively low morbidity at the donor-site after fibular graft. Most of patients have expressed satisfaction with both the functional and aesthetic outcomes despite the common complications of transient wound healing disorders and paraesthesia. Although the operation has caused a small but obvious decrease in stability and ankle function inspected by the Seated Excursion Balance Test (SEBT) and the Foot and Ankle Disability Index (FADI), the limitations is minimal in terms of quality of life and daily activities. The most effective way to compare the abundant data is to employ standardized test methods to assess the donor-site morbidity after fibula transplantation^28^.

Microsurgery technology, digital and 3D printing technology, and implantation technology enable vascularized iliac flap and fibular flap to reconstruct mandibular body defect to achieve a perfect functional repair effect. It not only shortens the operation time, restores the maxillofacial shape, but also achieves functional reconstruction and improves the patient’s QOL. It is recommended for patients with mandibular body defect to choose vascularized iliac bone flap for repair, while it is recommended for lesions with large defects or involving the condyle and chin to choose vascularized fibular flap to repair.

## Materials and methods

### Patients

A total of 61 patients with mandibular ameloblastoma who were admitted into the Department of Oral and Maxillofacial Surgery, First Affiliated Hospital of Zhengzhou University from September 2017 to November 2019 were selected, including 29 males and 32 females. There were 23 cases of iliac bone flaps and 38 cases of fibular flaps. All patients underwent extent mandibulectomy and free-flap (iliac bone flap or the fibula flap) reconstruction at the same time. The inclusion criteria were as follows: (1) The age ranged from 18 to 60 years old. (2) Preoperative design was carried out using 3D printing technology and digital technology. Intraoperative resection of the diseased mandible was performed, and the iliac bone flap or fibular flap technique was used for reconstruction and repair at the same time. Implant restorations were performed at 6–9 months after surgery. (3) No history of radiotherapy before surgery. (4) No recurrence of primary disease. (5) The observation period was more than 24 months. (6) None of the patients had cognitive impairment. All patients undertook the questionnaire on a voluntary basis.

### Ethical approval

This study was approved by the Ethics Committee of project of the First affiliated Hospital of Zhengzhou University (No. 2020-KY-230). All methods were performed in accordance with the relevant guidelines and regulations by the research ethics board. All participants provided written informed consent including publication of images.

### Sequential treatment steps

#### Fibular flap design and cutting

Preoperative resection of the diseased mandible was performed by utilizing digital technology. The shape of the mandible was restored using mirror technology. The number of osteotomy segments, the angle between the bone segments and the bone guide plate were designed after importing fibula data. The pre-bending preparation of the 2.0 reconstructed titanium plate was completed on the 3D head mold which was printed by 3D printing technology. The surgery is performed by two groups of doctors at the same time. One group underwent mandibular resection according to the extent of the lesion, all of which retained the articular disc. The condyle was retained depending on the specific situation, and the recipient arteries and veins were prepared at the same time. The other group has adopted Henry approach on the posterolateral side of the calf, and the fibula flap was routinely made. The skin island of the free fibular composite flap, the length of the fibula and the required muscles were designed depending on the extent of the defect. According to the preoperative digital design plan, the fibular flap was fixed with Swiss Sindis AO 2.0 prefabricated titanium plate before the pedicle was cut off. The peroneal artery was manually anastomosed with the facial artery or the superior thyroid artery with an 8–0 Prolene line. The microvascular anastomosis device was then used to anastomose the peroneal vein with the branch of the internal jugular veinto rebuild blood circulation.

#### Iliac bone flap design and cutting

The surgery is performed by two groups of doctors at the same time. One group underwent mandibular resection according to the extent of the lesion. The recipient arteries and veins were prepared at the same time. In the other group, along the marking line of the deep circumflex iliac artery and the inner and upper border of the skin flap, the incision was made to expose the deep circumflex iliac artery and vein, freeing in the direction of the iliac crest. The bone fragments were cut off according to the guide plate, and the deep circumflex iliac vessels were carefully protected to form an iliac musculocutaneous flap with the deep circumflex iliac artery and vein as the pedicle.

Implant surgery was performed 6–9 months after surgery. Porcelain crown restoration was performed 3–4 months after implanting. The adopted method was partial implant-supported fixed denture restoration or implant-supported overdenture restoration.

### Assessment tool

According to the standard procedures of the International Quality of Life Assessment (IQOLA), the Chinese versions of EORTC-QLQ-H&N and OHIP-14 were established (Gemert JV et al. 2015). It contains 35 common postoperative problems of patients with head and neck tumors, among which swallowing, language, mouth opening, diet, teeth and social contact are closely related to oral diseases.

EORTC-QLQ-H&N is specially designed for the QOL of head and neck cancer patients, and is currently one of the most commonly used questionnaires. It can be scored according to the scoring standard (1: no; 2: mild; 3: moderate; 4: severe). The higher the score, the worse the oral function of the patient.

OHIP-14 is a specific scale for oral and maxillofacial health, including seven aspects: functional limitations, physical pain, psychological discomfort, physical disability, psychological disability, social disability and handicap. The self-evaluation of each item in the scale is divided into 5 levels, with a score ranging from 0 to 100. The higher the total score, the worse the oral health.

### Assessment methods

The survey adopts the OHIP-14 questionnaire and the EORTC-QLQ-H&N scale, by a combination of doctors’ examination and questionnaire. The second and third questionnaires were filled out at follow-up visits at 6 and 24 months after surgery, and scored using the dedicated scoring manual provided by the questionnaire designer.

### Statistical analysis

The study has adopted the SPSS 22.0 statistical software to analyze the basic data of the two groups of patients. Independent samples t-test was performed on the EORTC-QLQ-H&N and OHIP-14 scores at two time points in each group. A two-sided p < 0.05 was considered as statistically significant difference.

## Data Availability

The datasets generated during and/or analysed during the current study are available from the corresponding author on reasonable request.
